# Annotations

**Published:** 1894-10-13

**Authors:** 


### Annotations.

### Death of Dr, Wendell Holmes.

The medical profession, as a body, does not probably contain a large number of persons who are familiar with the thoughts and philosophies of their great *confrère* who has just passed away in the eighty-sixth year of his age. Nevertheless, the writings of Dr. Oliver Wendell Holmes are eminently of the "readable" class, and, one would think, are peculiarly fitted to give refreshment and recreation to the somewhat abnormal literary appetite of the excessively "lectured," and book-and-newspaper ridden practitioner of medicine. Dr. Holmes combined in his own person the general practitioner, the man of science, and the philosopher; and, thinking of him, one wonders that such a combination is so rare in the medical profession, if indeed it be rare. The literary faculty one does not expect to find common in any profession, but science, practice, and philosophy ought to be the natural endowments of every man who has been duly bred to physic. In his youth Dr. Holmes contemplated the bar as his future calling, but after studying law for awhile he decided upon physic, and never afterwards regretted his choice. Undoubtedly physic has many advantages over law for the mind whose bent is towards science and philosophy, towards the larger humanities in fact. It is not every mind which is prepared at a moment's notice to undertake the task of "making the worse appear the better reason." "The Autocrat of the Breakfast Table" is probably the work of Dr. Holmes which will longest please the reading world; but lovers of literature will care to possess, and now and again to dip into, everything which he himself thought worthy of preservation. An amiable man was the Autocrat, a man of large and deep sympathies with his fellows. He knew no jealousy, either of the medical or literary kind. An ideal temper his was for a doctor. He will be missed and mourned by the whole English-speaking world. Of what other doctor could the same be said? Thinking of him as a practitioner of medicine, as a lecturer, and as a man, and of all the other members of our calling at the same moment, we cannot help breathing the ardent wish, *O si sic omnes.*

### The Japanese as Experimental Physiologists.

That the Westernisation (to coin a word) of the Japanese is much more than skin deep has been amply proved of late, not only by their warlike feats of arms in Korea, but also by their devotion to those scientific arts which make for "sweet reasonableness" and universal peace. One of the most ardent of modern physiological experimenters is Dr. Kitasato, a Jap, who is as well known in the scientific circles of Europe as he is in the far East. Dr. Kitasato has recently performed an important series of experiments with the object of elucidating the bacteriology and intimate pathology of the bubonic plague which is now raging in Hong Kong. The plague, as might have been anticipated, is discovered to be bacterial in causation, and displays in its progress the usual characteristics of bacterially produced diseases. It manifests a local lesion, the seat of lesion and the internal organs, as well as the blood, are all charged with the bacillus proper to the disease, the bacillus when inoculated directly, as from the bubo, or the blood, or one of the internal organs, reproduces itself in other animals, and its reproduction can be relied upon with equal certainty when inoculations are made from agar and other cultures. All this might have been looked for, and is nothing more than a sample of good laboratory work. But Dr. Kitasato has made his experiments with a thoroughness and philosophic insight which render them more than ordinarily interesting and valuable. Among other facts he has shown that the dust of rooms in which persons suffering from bubonic plague are confined, is charged with the specific bacteria to a greater or less extent, and that the disease may be effectually inoculated from it. Moreover, he has also demonstrated that the bacteria are destroyed in a few hours by exposure to the direct rays of the sun. This is much more than a mere approach to a philosophic comprehension, not only of the nature and progress of the disease, but also of its cause and of the rational means of preventing it on the largest possible scale. Dr. Kitasato's paper, which is published in the current number of the *Practitioner*, well deserves the study of his professional brethren of the West. It may be that science, rather than commerce or religion, is destined to carry the olive branch of peace and reasonableness into every quarter of the globe.

### Ireland for a Holiday.

Ireland is one of the most delightful countries for a holiday in all the world. So argues Mr. T. W. Russell, M.P., in a letter addressed to the Association for the Promotion of Home and Foreign Travel. The present writer has been over most of the ground described by Mr. Russell, and he can confirm every word which that sturdy Parliamentarian has written. Killarney, Mr. Russell says, is "beautiful." It is more than beautiful, it is incomparable. For a prolonged stay it is perhaps wanting in bracing qualities, but if bracing air be required there are parts of county Wicklow, within a score miles of Dublin, which are as dry and bracing as the Scotch Highlands about Balmoral. Mr. Russell is enthusiastic about Dublin Bay, and well he may be. From the Wicklow side of Dublin the views of the bay and of the blue sea of which it forms a part are exquisitely beautiful. As for the Irish people, the waiters, the jarvies, the peasantry, and all whom the traveller comes in contact with, they are, as is well known, the liveliest of the lively, and brimming over with wit. No man need spend a single dull day in Ireland. There is another incidental advantage which would result from holiday-making in Ireland. A large influx of generous English people would convince the stay-at-home Irish that the Sassenach has no particular kinship with the evil one; and would at the same time show the visitor how desirable it is, not only to retain Ireland as an integral portion of the British Empire, but also to bring about such a real union of hearts and interests as would make both Ireland and England pleased and contented with each other. Let the English tourist pay one visit to Ireland by all means. He will certainly want to pay a second and a third.

